# Mapping the multiscale neuroanatomy of *GRN*-related frontotemporal dementia using mode-based morphometry

**DOI:** 10.1016/j.nicl.2026.103994

**Published:** 2026-04-15

**Authors:** Enrico Premi, Giada Bianchetti, Valeria Bracca, Giulia Campana, Elena Gatti, Trang Cao, Alex Fornito, Valentina Cantoni, Sonia Bellini, Daniele Corbo, Mauro Magoni, Roberto Gasparotti, Roberta Ghidoni, Michela Pievani, Barbara Borroni

**Affiliations:** a Stroke Unit, ASST Spedali Civili, Brescia, Italy; b Laboratory of Alzheimer’s Neuroimaging and Epidemiology (LANE), IRCCS Istituto Centro San Giovanni di Dio Fatebenefratelli, Brescia, Italy; c Department of Molecular and Translational Medicine, University of Brescia, Brescia, Italy; d Department of Clinical and Experimental Sciences, University of Brescia, Italy; e The Turner Institute for Brain and Mental Health, School of Psychological Sciences, and Monash Biomedical Imaging, Monash University, Clayton, Victoria, Australia; f Molecular Markers Laboratory, IRCCS Istituto Centro San Giovanni di Dio Fatebenefratelli, Brescia, Italy; g Department of Medical Surgical Specialties, Radiological Sciences and Public Health, University of Brescia, Brescia, Italy

**Keywords:** Mode-based morphometry, Surface-based morphometry, GRN, Asymmetry, Frontotemporal dementia

## Abstract

Background: Individuals carrying Progranulin (*GRN*) mutations show asymmetrical grey matter atrophy, which could be used for early detection in the long asymptomatic phase. To capture these alterations, we employed both conventional Surface-Based Morphometry (SBM) and Mode-Based Morphometry (MBM). While the former provides high-resolution, location-specific estimates of cortical thickness (CT) differences, the latter has recently been introduced as a novel framework that decomposes CT maps into geometric eigenmodes, allowing a multiscale characterization of brain structural variability. Using both approaches enables the detection of complementary aspects of *GRN*-related neurodegeneration across spatial scales.

Methods: SBM and MBM were applied to CT maps to quantify structural alterations in individuals, 15 presymptomatic and 27 symptomatic, compared to 19 healthy controls (HC). SBM was used to assess vertex-wise CT differences, whereas MBM was used to decompose individual CT maps into geometric eigenmodes and quantify alterations across spatial scales. From both pipelines asymmetry indices (SBM-AI and MBM-AI) were computed. Associations between SBM/MBM-derived measures and domain-specific cognitive performance as well as global disease severity scores were then assessed.

Results: Compared with HC, symptomatic *GRN* showed significant alterations in seven eigenmodes in the left hemisphere, while only two modes contributed to CT differences in the right hemisphere. For MBM-AI and SBM-AI symptomatic *GRN* exhibited significantly different values compared to HC and presymptomatic *GRN* (*p* < 0.001). Although both asymmetry indices showed significant differences across disease stages (*p* = 1.3 × 10^−5^ SBM-AI; *p* = 3.5 × 10^−5^ MBM-AI), only the MBM-AI revealed a U-shaped trajectory across disease progression, characterized by an early increase in asymmetry followed by a partial re-symmetrisation in later stages.

Conclusions: MBM revealed multiscale cortical alterations in symptomatic *GRN* mutation carriers, capturing both large-scale hemispheric differences and more localized regional variations in CT that are less apparent with conventional SBM. These findings indicate that *GRN*-related neurodegeneration involves complex spatial pattern across multiple anatomical scales. Brain asymmetry remains a core hallmark of *GRN*-related pathology, supporting the use of asymmetry indices (derived from both SBM and MBM) as potential markers of disease progression at the symptomatic stage.

## Highlights

• MBM allows a multiscale characterization of brain structural variability.
• SBM and MBM detect complementary aspects of *GRN*-related neurodegeneration across spatial scales.
• Brain asymmetry remains a core sign of *GRN* disease and a potential biomarker of disease progression.

## 1 Introduction

Frontotemporal Dementia (FTD) is a leading cause of early-onset dementia worldwide, with genetic forms accounting for nearly 30% of cases (Warren et al., 2013a). The most frequently implicated genes are C9 open reading frame 72 (*C9orf72*), microtubule-associated protein Tau (*MAPT*) and Progranulin (*GRN*) (Moore et al., 2020). Among these, *GRN* mutation carriers display distinctive patterns of disease progression, as well as clinical and radiological phenotypes (Rohrer et al., 2010, Whitwell et al., 2012, Rohrer et al., 2015). *GRN* disease is characterized by asymmetrical grey matter atrophy, primarily involving the left, frontal, posterior temporal, and inferior parietal regions (Samra et al., 2023). Individuals may remain asymptomatic for decades until sudden clinical and radiological changes arise with a rapid conversion toward dementia (Cash et al., 2018, Poos et al., 2022, Staffaroni et al., 2022, Staffaroni et al., 2020). Behavioral variant Frontotemporal Dementia (bvFTD), non-fluent Primary Progressive Aphasia (nfPPA) and Corticobasal Syndrome (CBS) are the most common phenotypes (van Swieten and Heutink, 2008). Genetic modifiers, such as the TMEM106B haplotype, cognitive reserve, and other environmental factors, can influence the age of disease onset, the rate of progression, and the clinical-cognitive expression of the disease (Cruchaga et al., 2011, Gaweda-Walerych et al., 2025, Mirza et al., 2025, Premi et al., 2017, Premi et al., 2014, Premi et al., 2013).

Given the long asymptomatic phase that characterizes genetic dementia, the identification of early disease markers remains an open area of research. In this vein, identifying robust and sensitive MRI markers for evaluating the radiological continuum of the FTD spectrum may support clinicians in prognostic assessments, enable earlier detection of disease progression, and guide personalised management strategies. Numerous imaging studies have investigated both grey and white matter involvement in presymptomatic *GRN* mutation carriers (Borroni et al., 2008, McKenna et al., 2022, Panman et al., 2021, Panman et al., 2019, Popuri et al., 2018), reporting mixed findings in analyses of cortical thickness compared to healthy controls (Pievani et al., 2014), with the largest study on this topic reporting no significant differences in cortical thickness in *GRN* mutation carriers (Borrego-Écija et al., 2021). These findings remain insufficient for early and reliable disease monitoring, prompting growing attention to the characterization of other morphometric features in this genetic group.

Grey matter asymmetry, expressed as an Asymmetry Index (AI), is now gaining increasing interest as a potential disease biomarker (Borrego-Ecija et al., 2024, Whitwell et al., 2012). Traditionally, Voxel-Based Morphometry (VBM) and Surface-Based Morphometry (SBM) approaches have been used to calculate AI as the difference between left (LH) and right (RH) hemisphere volume or thickness, showing significant differences almost ten years before the expected clinical onset in asymptomatic carriers (Borrego-Ecija et al., 2024). However, evidence remain scarce and these approaches are limited to detecting anatomical differences at a single spatial resolution scale.

Recently, Cao et al. introduced Mode-Based Morphometry (MBM), a multiscale method that may offer novel insights into the complex organization of the human brain (Cao et al., 2024). MBM decomposes cortical thickness using *eigenmodes* derived from the shape of the cortex. These eigenmode approximate fundamental resonant modes of brain structure, which constraint dynamical processes unfolding in the brain (Pang et al., 2023). By decomposing a map of cortical thickness changes using geometric eigenmodes, MBM offers a spectral characterization of case-control differences across different spatial scales, potentially providing complementary information on the underlying pathological condition and its intrinsic disruptive patterns. In particular, lower-order eigenmodes can capture geometric properties at a coarser scales, such as variations along major functional/structural gradients (e.g., medial–lateral, anterior–posterior, and dorsal–ventral axes), whereas higher-order eigenmodes are sensitive to more localized variations (e.g., fine-grained gyral/subregional changes) (Pang et al., 2023). MBM has demonstrated comparable or superior validity and consistency when compared with classical SBM, especially on noisy data (Cao et al., 2024). Geometric eigenmodes have also been used to study cortical symmetries, and can offer more reliable and sensitive measures than traditional morphological metrics such as volume, cortical thickness, surface area (Chen et al., 2024). In neurodegenerative diseases characterized by cortical asymmetry and brain changes at different scales, such as FTD, this approach may offer a powerful mean to identity subtle structural alterations.

In the present study, we aimed to: a) characterize cortical differences between symptomatic and asymptomatic *GRN* mutation carriers and healthy controls with MBM compared to the conventional SBM approach; b) compare the performance of MBM-derived AI with conventional SBM-based AI; and c) explore the relationship between SBM/MBM cortical alterations and cognitive and clinical measures.

## 2 Methods

### 2.1 Participants

A total of 81 individuals were initially enrolled for the present study, all recruited at the Centre for Neurodegenerative Disorders, University of Brescia (Brescia, Italy), between November 2015 and March 2025. Participants included 26 healthy controls (HC)**,** 28 presymptomatic GRN mutation carriers, and 27 symptomatic GRN mutation carriers. All *GRN* mutation carriers were confirmed by genetic testing.

Given the substantial age differences between symptomatic and presymptomatic individuals inherent to genetic cohorts, we observed a limited overlap in age distributions across groups in the initial sample (mean age: 63 ± 8 years for symptomatic carriers, 49 ± 11 years for presymptomatic carriers). Under these conditions, adjusting for age as a covariate could lead to unreliable estimates due to extrapolation beyond the observed data. Therefore, to minimize age-related confounding, we adopted a conservative approach by matching groups at the sample level. Specifically, participants were excluded to achieve comparable age distributions across groups.

This procedure led to the exclusion of younger HC and presymptomatic individuals, resulting in a final cohort of 61 participants, distributed as follows: 19 HC, 15 presymptomatic *GRN* mutation carriers and 27 symptomatic *GRN* mutation carriers. Within the group of symptomatic GRN mutation carriers, 18 individuals had a clinical diagnosis of nfPPA, 6 individuals with bvFTD, 1 with the semantic variant of PPA (svPPA), 1 with CBS, and 1 with posterior cortical atrophy (PCA).

Symptomatic and presymptomatic participants underwent a clinical and cognitive evaluation, which included the Clinical Dementia Staging Instrument plus National Alzheimer's Coordinating Center Behavior and Language Domains – Global Score (CDR plus NACC) (Miyagawa et al., 2020), which assesses disease severity across multiple cognitive, functional, behavioural, and language domains, and yields a Global CDR score used to classify overall disease severity (0 = no dementia, 0.5 = very mild impairment, 1 = mild dementia, 2 = moderate dementia, 3 = severe dementia). Symptomatic patients were also tested with the Trail Making Test (parts A and B), which measures processing speed, visual-conceptual tracking, mental flexibility, and executive functioning (Corrigan and Hinkeldey, 1987). For both parts, the outcome measure is the completion time in seconds, with longer times indicating poorer performance. Written informed consent was obtained from all participants in accordance with the Declaration of Helsinki. The study protocol was approved by the local Ethics Committee.

### 2.2 MRI acquisition

Each participant underwent a structural brain MRI using a Siemens Skyra 3T scanner with 64 channels at the Neuroradiology Unit of the ASST Spedali Civili in Brescia. The protocol included a volumetric T1-weighted scan acquired with a magnetization-prepared rapid gradient echo (MPRAGE) sequence (repetition time [TR] = 2000 ms, echo time [TE] = 2.92 ms, voxel size = 1.1 mm isotropic, acquisition matrix = 256 × 256).

### 2.3 Surface-Based Morphometry (SBM) analysis

All 3D T1-weighted MRI scans underwent visual quality control to assess the presence of motion or other image artifacts. Images were then processed using the *recon-all* pipeline in FreeSurfer v7.4.1 (https://surfer.nmr.mgh.harvard.edu/), which performs fully automated cortical and subcortical segmentation and surface reconstruction (Fischl, 2012). The preprocessing steps comprised motion correction, bias field correction, skull stripping, and both linear and non-linear Talairach transformations to the MNI305 standard space. Subsequent processing included intensity normalization, segmentation of white matter and subcortical structures, tessellation of the grey-white matter interface, correction of topological defects, and surface deformation driven by local intensity gradients. Finally, surface inflation, registration to a spherical atlas, and cortical parcellation were performed to generate subject-specific surface-based morphometric data (Fischl, 2012). The quality of FreeSurfer outputs was assessed using Qoala-T (Klapwijk et al., 2019), and all cases were confirmed as suitable for analysis through visual inspection. No participant was excluded based on quality control.

Following reconstruction, cortical thickness (CT) values were mapped separately for the LH and RH onto the *fsaverage* template with 163,842 vertices using *mri_surf2surf*, and subsequently preprocessed across subjects with *mris_preproc*. Surface data were smoothed with a 10 mm FWHM Gaussian kernel. Group-level statistical analyses were performed with the general linear model (GLM) implemented in FreeSurfer (*mri_glmfit*). The design matrix encoding group membership was specified through a FreeSurfer Group Descriptor File (FSGD), and the following contrasts were tested: (i) symptomatic *GRN* mutation carriers vs. HC, and (ii) presymptomatic *GRN* mutation carriers vs. HC. Multiple comparisons adjustment was performed via cluster-wise correction using *mri_glmfit-sim*, with a vertex-wise threshold of *p* < 0.001 and a cluster-wise significance level of *p* = 0.05.

### 2.4 Mode-Based Morphometry (MBM) analysis

MBM is a computational framework that decomposes anatomical variations into spatial frequency components using the fundamental resonant modes––eigenmodes––of brain anatomy. In this case, we used eigenmodes extracted from the geometry of the cortical surface mesh, as they are reliable, robust, and can account for diverse features of brain organization (Cao et al., 2024, Pang et al., 2023).

MBM enables the characterization of group differences across multiple spatial scales through the analysis of the contribution of each eigenmode to anatomical variation. In the present study, MBM was applied using the publicly available toolbox described in (Cao et al., 2024) and available at https://github.com/NSBLab/MBM.

CT maps obtained from FreeSurfer as described in Section 2.3 were used as input, along with a binary cortical mask and a surface mesh**,** both common for all subjects, generated via custom R-based in-house scripts. Specifically, the mask was created by extracting cortical vertices coordinates from the *cortex.label* file for each hemisphere and assigning a value equal to 1 to those belonging to cortex (0 to others), matching the *fsaverage* mesh resolution (163842 vertices). The surface mesh was derived by converting the *pial* surface of the *fsaverage* subject into *vtk* format and subsequently used for both eigenmode computation and result visualization within MBM.

The geometric eigenmodes were obtained by solving the eigendecomposition of the Laplace–Beltrami Operator (LBO), also known as the Helmholtz equation, on the cortical surface:

Δψ=-λψ

where Δ is the LBO, and the solution $\psi =\left\{\psi_{1},\psi_{2},\cdots \right\}$ is the family of geometric eigenmodes associated with eigenvalues $\lambda =\left\{\lambda_{1},\lambda_{2},\cdots \right\}$. These eigenmodes form a spatially-ordered orthonormal basis that enables multiscale decomposition of cortical anatomy (Atasoy et al., 2016, Gabay and Robinson, 2017), analogous to a Fourier decomposition. A total of 150 eigenmodes of the fsaverage surface were included in the analysis to capture spatial differences across a broad range of anatomical scales, as in (Cao et al., 2024). In the MBM framework, each individual’s cortical anatomy is represented by a vector of modal loading coefficients (β), which quantify the contribution of each eigenmode to that subject’s CT map. These β coefficients are the parameters on which statistical inference is performed, as they provide a multiscale, mode-specific description of anatomical variation.

Group-level statistical analyses were performed separately for each hemisphere and for the following comparisons: (1) symptomatic *GRN* mutation carriers vs. HC, and (2) presymptomatic *GRN* carriers vs. HC. Analyses were conducted via the graphical interface *MBM_toolbox.mlapp* in MATLAB. A two-sample *t*-test was performed for each comparison using a design matrix encoding group membership. The number of non-parametric permutations for multiple comparisons was set to 10000, and statistical significance was assessed using a *p*-value threshold of 0.05.

### 2.5 Quantification of asymmetry index (AI)

Given that FTD is characterized by asymmetric changes, we investigated the sensitivity of asymmetry indices derived from MBM and conventional approach, respectively, in distinguishing between clinical groups.

#### 2.5.1 Conventional asymmetry index (SBM-AI)

A standard anatomical AI was computed based on CT values obtained from SBM preprocessing. To this aim, we used the significance map obtained from the contrast between HC and symptomatic participants in SBM analysis (section 2.3) and selected the dominant hemisphere (i.e., with the most pronounced effect, typically the left). The hemispheric map was binarized (threshold: *p* < 0.05), flipped and applied to the contralateral hemisphere using FreeSurfer’s cross-hemispheric mapping to create a symmetric bilateral region of interest, allowing the extraction of mean CT values from both hemispheres on the same anatomical regions. The symmetric bilateral mask is illustrated in Supplementary Fig. 2.

For each subject, the mean CT within this mask was computed for each hemisphere (LH and RH), and the AI was defined as:

$$\mathrm{AI}=\left|\frac{CT_{\mathrm{RH}}-CT_{\mathrm{LH}}}{0.5\times (CT_{\mathrm{RH}}+CT_{\mathrm{LH}})}\right|$$

where $CT_{\mathrm{RH}}$ and $CT_{\mathrm{LH}}$ represent the mean CT values in the RH and LH, respectively.

Differences in conventional SBM-AI across groups were assessed with the Kruskal–Wallis test, given that all distributions deviated from normality and variances were not homogeneous. Pairwise comparisons were subsequently carried out using Dunn’s post-hoc tests with Benjamini-Hochberg (BH) correction.

#### 2.5.2 MBM asymmetry index (MBM-AI)

To evaluate anatomical asymmetry across spatial scales, we performed a subject-level decomposition of CT maps into geometric eigenmodes. For each individual and hemisphere, CT data were first masked to exclude non-cortical vertices and then projected onto the set of the 150 eigenmodes derived from the *fsaverage* pial surface (as described in section 2.4) using a custom MATLAB-based script. The projection coefficients (modal weights, β) were obtained by solving a least-squares eigen-decomposition, such that each CT map *f* was approximated as a linear combination of eigenmodes $\psi_{k}$:

$$f\approx \sum_{k=1}^{N}\beta_{k}\psi_{k}$$

where $\beta_{k}$ represents the contribution of the k-th eigenmode.

Interhemispheric dissimilarity was then assessed directly from the modal weights. For each subject, we extracted the vector of β coefficients corresponding to the first *N* modes in the LH and RH and computed the Pearson correlation coefficient between them. In this analysis, only the first *N* = 25 modes, corresponding to the first four eigengroups (Chen et al., 2022, Robinson et al., 2016) were included. These modes capture the majority of variance in cortical thickness at macroscopic and sub-regional spatial scales, while higher-order modes predominantly reflect fine-grained, high-frequency spatial patterns that are more susceptible to noise and inter-individual variability. Restricting the analysis to the first four eigengroups, i.e. 25 modes, therefore provides a more robust and biologically interpretable estimate of interhemispheric asymmetry.

Individual-level interhemispheric asymmetry was quantified as the absolute value of the following correlation:

$$\left|corr(\left[\beta_{1}^{\mathrm{LH}},\cdots ,\beta_{N}^{\mathrm{LH}}\right],\left[\beta_{1}^{\mathrm{RH}},\cdots ,\beta_{N}^{\mathrm{RH}}\right])\right|$$

Lower values of this index indicate greater asymmetry, reflecting dissimilarity in the eigenmode-based decomposition of cortical thickness profiles across hemispheres, independent of correlation sign. Group differences in MBM-AI among HC, presymptomatic, and symptomatic *GRN* mutation carriers were evaluated using the non-parametric Kruskal–Wallis test, as data showed deviations from normality in all groups (Shapiro–Wilk test) and significant heterogeneity of variances (Levene’s test, *p* < 0.001). Post-hoc comparisons were performed with Dunn’s tests using BH correction for multiple comparisons.

### 2.6 Analysis of the relationship between AI and cognitive features

To investigate the association between cortical asymmetry and cognitive features, we examined the distribution of SBM-AI and MBM-AI, respectively, across levels of the CDR plus NACC as well as their correlation with TMT-A and TMT-B. Since CDR is designed to stage cognitive impairment in the dementia spectrum, presymptomatic and symptomatic individuals were grouped together, whereas HC were treated as a separate reference group. Group differences in the AI were assessed using Kruskal-Wallis test with CDR plus NACC group as the between-subjects factor. Post-hoc pairwise comparisons were performed using Dunn’s test with BH correction for multiple comparisons. For TMT-A and TMT-B, we computed Spearman’s rank correlation coefficients (ρ) and corresponding *p*-values, restricting the analysis to symptomatic individuals.

### 2.7 Analysis of the relationship between SBM/MBM cortical alterations and cognitive features

To assess if the patterns of CT variations (reflected by SBM CT maps as well as the eigenmode coefficients obtained from MBM analysis) explained individual differences in clinical severity and cognitive performance, GLM tests (R, version 4.3.2) were built with TMT-A, TMT-B and CDR plus NACC as dependent variables. Predictors included the modal weights β of the significant eigenmodes extracted separately for the LH and RH. t-values and corresponding significance *p* values were evaluated to identify components significantly associated with cognitive features.

For the SBM analysis, the relationship between vertex-wise CT and cognitive variables was assessed using a correlation design implemented in FreeSurfer (v7.4.1) with the *mri_glmfit* tool. Statistical significance was determined using cluster-wise correction for multiple comparisons, with a cluster-forming threshold of *p* < 0.001, a minimum cluster area of 100 mm^2^, and a cluster-wise corrected significance level of *p* < 0.05. Negative correlations indicate that higher clinical severity (higher CDR-Global Score) is associated with reduced cortical thickness.

## 3 Results

### 3.1 Clinical and demographic features of participants

Table 1 illustrates demographic and clinical features of the 61 participants (HC = 19, presymptomatic *GRN* carriers = 15, symptomatic *GRN* carriers = 27). HC and presymptomatic *GRN* carriers were slightly younger than symptomatic, although the difference was not statistically significant (*p* = 0.14). The groups were also balanced for sex distribution (*p* = 0.3). *GRN* mutation carriers showed significantly higher educational attainment compared with HC (overall *p* = 0.020; symptomatic vs HC *p* = 0.022). As expected, symptomatic *GRN* carriers were characterized by greater disease severity than presymptomatic carriers (*p* < 0.001).

**Table 1:** Demographic and main clinical characteristics of the study groups.

Variable	HC N = 19	Presymptomatic N = 15	Symptomatic N = 27	p-value
Age	61 ± 12 (39 – 80)	57 ± 8 (46 – 75)	63 ± 8 (50 – 79)	0.14
Sex [females]	14 (74%)	10 (67%)	14 (52%)	0.3
Education	8.5 ± 3.3 (5.0 – 17.0)	11.7 ± 4.5 (8.0 – 19.0)	11.8 ± 4.2 (4.0 – 19.0)^#^	0.020 (*)
CDR plus NACC	−	0.1 ± 0.2 (0.0 – 0.5)	1.6 ± 0.8 (0.5 – 3.0)	< 0.001 (***)
TMT-A	−	−	132 ± 116 (19 – 300)	
TMT-B	−	−	414 ± 155 (55 – 500)	

Values are presented as mean ± standard deviation (min–max) for continuous variables and as number (%) for categorical variables. Group comparisons were performed using one-way analysis of variance (ANOVA) for continuous variables and Pearson’s Chi-squared test for categorical variables. Symbols denote significance levels: * p < 0.05; *** p < 0.001; ^#^ p-adj < 0.05 vs. HC.

### 3.2 Multiscale mapping of cortical thickness alterations in GRN mutation carriers

**Fig. 1:**
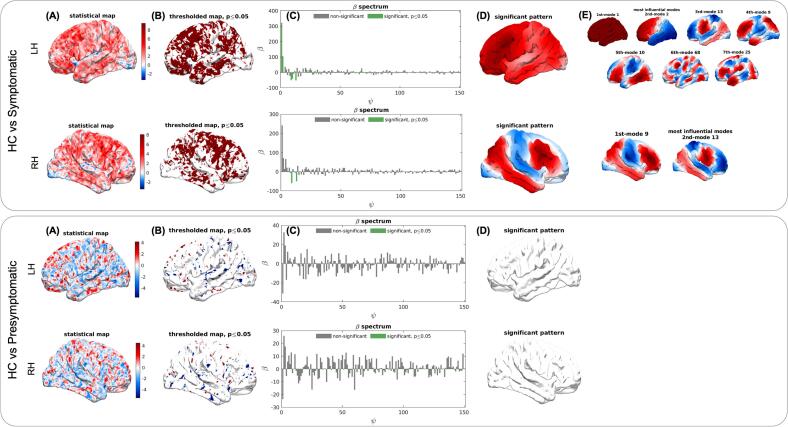
MBM analysis of CT differences between HC and symptomatic (upper panel) and presymptomatic (lower panel) *GRN* mutation carriers. (A) Unthresholded *t*-map. Negative-zero-positive values are colored as blue-white-red, with positive values indicating reduced thickness in *GRN* carriers. The thresholded *t*-map at *p* ≤ 0.05 are shown in (B). Red and blue denote significantly thinner CT in *GRN* carriers and HC, respectively. The β spectrum is reported in (C), with β’s of the significant modes colored in green. The significant pattern obtained by combining the significant modes weighted by their β is reported in (D) and the most influential modes for each contrast are represented in (E). (For interpretation of the references to colour in this figure legend, the reader is referred to the web version of this article.)

Fig. 1 illustrates the results of the MBM analysis for the comparison between symptomatic *GRN* mutation carriers and HC (upper panel) and presymptomatic mutation carriers vs. HC (lower panel). For each contrast and hemisphere (LH and RH, respectively), unthresholded (A) and thresholded (*p* < 0.05; B) statistical *t*-maps of CT differences are shown, together with the corresponding β spectra (indicating the contribution of each eigenmode; C), the reconstructed spatial patterns from significant modes (*p* < 0.05; D), and the most influential modes driving the observed group-level effects (E).

Significant eigenmodes were identified for the comparison between symptomatic *GRN* carriers and HC. In the LH, seven modes reached significance (modes 1, 2, 13, 9, 10, 68, and 25, ordered by decreasing |β|), while in the RH only two modes (9 and 13) contributed to CT differences. The majority of significant eigenmodes corresponded to low-frequency, coarse-scale mode for both LH and RH, indicating that CT differences are mostly explained by global, large-scale reductions rather than focal effects. Mode 1, which contributed to CT differences only for the LH, displayed a globally uniform pattern, consistent with a pattern of widespread cortical thinning in the dominant hemisphere. Mode 2, which was also specific to the LH, corresponded to an anterior–posterior gradient, indicating greater frontal than posterior thinning. Mode 13, which was observed bilaterally, was strongly weighted on the insula and additionally included temporoparietal regions, corresponding to the typical pattern of atrophy observed in FTD. Modes 9 and 10 involved frontal, parietal and lateral temporal areas, consistent with networks supporting executive and semantic functions. The remaining modes contributing to left CT differences (68 and 25) exhibited more fragmented, fine-grained patterns, reflecting localized structural variations that are less easily mapped onto specific regions or neuroanatomical systems. In the RH, eigenmodes contributing to CT differences were identified at intermediate scales (i.e., neither the lowest-order nor the finer-grained modes were significant), indicating more localized effects compared to the LH. Finally, no significant eigenmodes were identified for the comparison between presymptomatic *GRN* carriers and HC.

### 3.3 Surface-based mapping of CT alterations in GRN mutation carriers

The results of the conventional SBM analysis examining CT differences between groups is reported in Supplementary Fig. 1. A significant cortical thinning was observed only for the comparison between symptomatic *GRN* carriers and HC, mapping predominantly to the LH in frontal (superior, caudal middle frontal, and orbitofrontal gyrus), temporal (middle and inferior temporal gyrus), and parietal (supramarginal gyrus) regions. In the RH, effects were mainly localized in the frontal cortex (precentral, superior frontal, caudal middle frontal and pars orbitalis gyrus), with smaller clusters in the temporo-parietal cortex (precuneus, inferior and superior parietal gyrus). These findings were significant at a vertex-wise cluster-forming threshold of *p* < 0.001 and survived cluster-wise correction at *p* < 0.05. In contrast, the comparison between presymptomatic *GRN* carriers and HC (Suppl. Fig. 1B) did not reveal any significant CT alterations, even with the more liberal statistical threshold of *p* < 0.001, uncorrected for multiple comparisons.

### 3.4 Assessment of cortical asymmetry in GRN mutation carriers

**Fig. 2:**
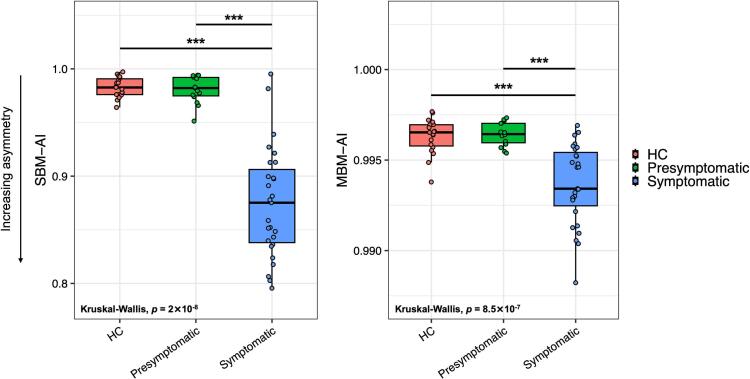
Distribution of the conventional asymmetry index (SBM-AI, left panel) and MBM-AI (right panel) according to disease stage. The AI indexes are shown for healthy controls (HC, red), presymptomatic *GRN* carriers (green), and symptomatic *GRN* carriers (blue). Values closer to 1 indicate greater interhemispheric similarity, while lower values reflect increasing asymmetry. Statistical analyses (Kruskal–Wallis test followed by Dunn’s post-hoc tests with Benjamini-Hochberg correction) are reported on the graphs (*** stands for *p* < 0.001). (For interpretation of the references to colour in this figure legend, the reader is referred to the web version of this article.)

Fig. 2 depicts the distribution of asymmetry indices in HC (red), presymptomatic (green), and symptomatic (blue) *GRN* carriers. Values closer to 1 indicate greater interhemispheric similarity, whereas lower values reflect increasing asymmetry.

Both approaches revealed a consistent trend, with comparable values between HC and presymptomatic *GRN* carriers and a marked increase in asymmetry in symptomatic individuals. For the MBM-derived asymmetry index, mean values were close to 1 in both HC (MBM-AI_HC_: 0.996 ± 0.0010) and presymptomatic carriers (MBM-AI_Presympt:_ 0.996 ± 0.0006), while symptomatic carriers showed reduced interhemispheric similarity (MBM-AI_Sympt:_ 0.994 ± 0.0022).

Statistical analyses confirmed a significant main effect of group (Kruskal–Wallis, *p* = 8.5 × 10^−7^), with post hoc tests (corrected for multiple comparisons) indicating lower MBM-AI values in symptomatic carriers compared with both HC (*p* < 0.001) and presymptomatic carriers (*p* < 0.001), and no significant difference between HC and presymptomatic groups. Similarly, for the conventional SBM-derived asymmetry index, mean values were close to 1 in HC (SBM-AI_HC_: 0.983 ± 0.009) and presymptomatic carriers (SBM-AI_Presympt:_ 0.981 ± 0.013), but markedly lower in symptomatic individuals (SBM-AI_Sympt:_ 0.895 ± 0.052). The group effect was significant (*p* = 2 × 10^−8^), with post hoc comparisons confirming reduced indices in symptomatic carriers versus both HC (*p* < 0.001) and presymptomatic carriers (*p* < 0.001), and no significant difference between HC and presymptomatic carriers. When the different clinical phenotypes were considered (avPPA and bvFTD), groups display lower SBM-AI and MBM-AI values compared to healthy controls (HC) and presymptomatic carriers, consistent with greater cerebral asymmetry in symptomatic disease. Notably, avPPA and bvFTD do not differ significantly from each other, suggesting that the degree of captured asymmetry is not strongly driven by clinical phenotype per se.

Effect size estimates (Cohen’s d) for all pairwise comparisons are reported in Supplementary Tables 1 (MBM-AI) and 2 (SBM-AI). An additional correlation-based SBM asymmetry index was computed to confirm these findings (see Supplementary Section “Correlation-based SBM Asymmetry Index” and Supplementary Fig. 3), which showed a consistent pattern of increased asymmetry in symptomatic *GRN* carriers.

### 3.5 Correlation of asymmetry indexes with disease severity

**Fig. 3:**
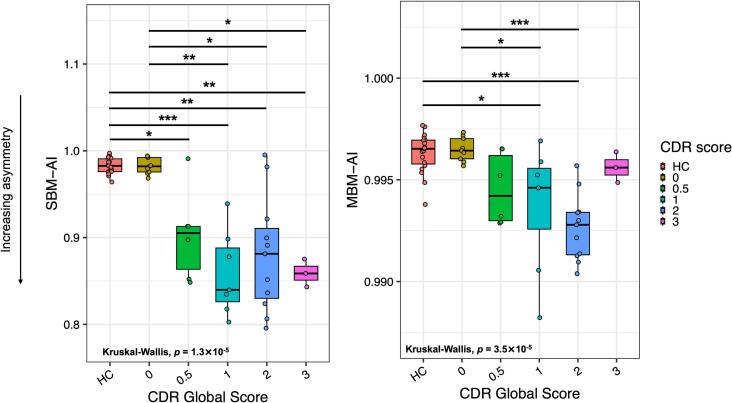
Distribution of conventional (SBM-AI, on the left) and MBM-derived asymmetry index (MBM-AI, on the right) according to disease severity. The box plots show AI for healthy controls (HC, red) and *GRN* mutation carriers stratified by global CDR score: 0 (yellow), 0.5 (green), 1 (cyan), 2 (blue), and 3 (magenta). Lower AI values indicate greater asymmetry. Statistical differences between groups were assessed using Kruskal-Wallis test, with post-hoc comparisons indicated by asterisks (* stands for *p* < 0.05; ** for *p* < 0.01; *** for *p* < 0.001). (For interpretation of the references to colour in this figure legend, the reader is referred to the web version of this article.)

To evaluate the relationship between asymmetry indexes and disease severity, we first examined their distribution across disease severity groups defined by the CDR plus NACC (Fig. 3). Both indices showed significant differences across CDR-defined groups (Fig. 3), with the model revealing a significant group effect (*p* = 3.5 × 10^−5^ for MBM-AI and *p* = 1.3 × 10^−5^ for SBM-AI, respectively).

For the MBM-derived asymmetry index, healthy controls (HC; reference group, in red) displayed the highest interhemispheric similarity (0.996 ± 0.0010), with comparable values in presymptomatic GRN mutation carriers with CDR = 0 (N = 9, in yellow; 0.996 ± 0.0006). A modest, non-significant reduction was observed at CDR = 0.5 (0.995 ± 0.0018), while a significant decrease emerged at CDR = 1 (N = 7; 0.994 ± 0.0031; *p* = 0.044 vs HC; *p* = 0.049 vs CDR = 0). At the moderate stage (CDR = 2, N = 11), MBM-AI values further decreased (0.993 ± 0.0016), significantly differing from both HC and presymptomatic groups (*p* < 0.001). Interestingly, at the severe stage (CDR = 3, N = 3), MBM-AI values returned to levels comparable to those of HC and presymptomatic carriers (0.996 ± 0.0007).

A similar pattern was observed for the conventional SBM-derived asymmetry index. Mean values were close to 1 in HC (0.983 ± 0.0091) and presymptomatic carriers (0.983 ± 0.0091, CDR = 0), with a significant reduction already evident at the earliest symptomatic stage (CDR = 0.5; 0.902 ± 0.052; *p* = 0.036 vs HC; *p* = 0.002 vs CDR = 0). At CDR = 1, asymmetry increased further (0.859 ± 0.049), though not significantly compared with CDR = 0.5. The most marked disruption was observed at the moderate stage (CDR = 2; 0.880 ± 0.066), significantly different from both HC (*p* = 0.003) and CDR = 0 (*p* = 0.010). At the severe stage (CDR = 3), SBM-AI values remained lower than those of HC and presymptomatic carriers (0.859 ± 0.016).

Effect size estimates (Cohen’s d) for all pairwise comparisons are reported in Supplementary Tables 3 (MBM-AI) and 4 (SBM-AI).

Additionally, we explored the Spearman correlation between asymmetry and executive function as measured by the Trail Making Test part A and B, respectively, which revealed no significant associations between asymmetry indexes and TMT performance.

### 3.6 Association between cognitive features and SBM/MBM CT alterations

For the MBM analysis, the modal weights, β, of the significant eigenmodes identified in Section 3.2 (LH: modes 1, 2, 9, 10, 13, 25, 68; RH: modes 9, 13) were included as predictors in the assessment of associations with clinical severity (CDR plus NACC) and cognitive performance (TMT-A and TMT-B). Regarding clinical severity, only the RH mode 9 showed a significant association with the CDR plus NACC score (*t* = 2.306, *p* = 0.029), whereas all other modes were not significantly related to disease severity. Significant associations with TMT-A emerged for several LH modes: mode 1 (*t* = 3.418, *p* = 0.006), mode 9 (*t* = 4.235, *p* = 0.001), mode 13 (*t* = 5.078, *p* < 0.001), and mode 25 (*t* = –2.713, *p* = 0.020). Other LH modes (2, 10, 68) and RH modes (9, 13) did not reach statistical significance (all *p* > 0.05). No significant associations were observed between any mode and TMT-B performance.

The negative (where the greater disease severity was related to lower CT) association between CDR plus NACC and CT SBM demonstrated three clusters (>100 mm^2^) localized in the LH: one in the left middle cingulate cortex, one in the left precuneus and one in the left cuneus region (all with *p* = 0.0002). In the RH, one cluster was identified in the middle frontal region (*p* = 0.0002) and another in the supramarginal region (*p* = 0.0002). In contrast, neither part A nor B of the TMT showed significant correlations with CT.

## 4 Discussion

In the present study we applied MBM, a recently developed approach for characterizing multiscale case-control differences in neuroanatomy, to a cohort of subjects carrying *GRN* mutations (presymptomatic and patients with FTD) compared to a group of healthy controls. Leveraging this multiscale method, we studied cortical thickness changes resulting from the contributions of different eigenmode that represent the intrinsic resonant modes of the brain at different spatial resolution scales, providing complementary information to conventional approaches like SBM (Chen et al., 2024, Pang et al., 2023). As a potentially distinctive neuroanatomical feature of *GRN*-related disease, we also investigated grey matter asymmetry with MBM-AI and its association with clinical and cognitive features, in line with previous literature data (Borrego-Ecija et al., 2024, Licata et al., 2020). This analysis showed that the MBM-AI index exhibits a U-shaped trajectory across clinical stages, reflecting disease progression but not cognitive decline. The pattern suggests that MBM-AI is particularly sensitive to structural asymmetry changes associated with neurodegeneration, while remaining largely independent of TMT-measured cognitive performance. Compared with the conventional SBM approach—which revealed predominant left-sided cortical thinning, particularly in the frontal, temporal, and parietal regions of symptomatic *GRN* carriers—the multiscale MBM analysis demonstrated additional, complementary patterns. Specifically, we observed greater involvement in the LH (seven significant eigenmodes) compared with the right (two eigenmodes). Examination of the significant eigenmodes revealed large-scale effects, ranging from widespread cortical involvement of the LH to anterior–posterior gradients with predominant anterior damage. Notably, one specific mode (mode 13) showed strong bilateral involvement of the insula, consistent with previous studies describing functional network impairment in *GRN* disease, particularly within the salience and language networks (Flagan et al., 2025). Interestingly, this focal insular involvement aligns well with the conceptual framework of “molecular nexopathies” proposed by Warren et al. for the different proteinopathies underlying FTD (Warren et al., 2013b). Unfortunately, no significant eigenmodes were identified for presymptomatic *GRN* carriers—consistent with previous structural studies, though likely also related to the small sample size (Borrego-Écija et al., 2021).

We initially hypothesized that the asymmetric pattern of *GRN*-related neuroanatomical involvement could serve as a potential early disease marker. For this reason, AIs were calculated from both conventional SBM and MBM-derived data (using the first 25 modes, which capture large-scale to sub-regional cortical variations and contains most of the significant modes when comparing CT changes between HC and symptomatic groups). Both indices were sensitive to the symptomatic phase of *GRN* disease, showing greater asymmetry in *GRN* carriers compared with HC. Although no significant differences were observed for presymptomatic carriers, a progressive increase in asymmetry was evident with disease severity, as measured by the CDR plus NACC score. Interestingly, while the SBM-AI was already altered in the mild stage (CDR = 0.5), the MBM-AI displayed a U-shaped pattern across disease stages (CDR = 0 to CDR = 3), showing increasing asymmetry in mild disease and a relative return toward symmetry in advanced stages (CDR = 3). Although reduced asymmetry at more advanced stages should be interpreted cautiously as only few cases were included in this group, we interpret this pattern as indicative of a homogeneous spread of damage across hemispheres rather than as evidence of clinical recovery or improvement. This interpretation is consistent with the known neuroanatomical progression of *GRN* disease, characterized by increasingly bilateral cortical involvement in advanced stages (Warren et al., 2013b, Whitwell et al., 2009).

While SBM correlations with executive measures were null, MBM eigenmodes revealed left-lateralized, multiscale associations with processing speed (TMT-A) spanning from global (mode 1) to progressively focal components (e.g., mode 9: fronto-temporo-parietal; mode 13: insular; mode 25: finer-grained frontal/temporal). These findings pertain to spatial-spectral features (eigenmodes), not to the asymmetry index, and support a multiscale, network-level account of executive slowing in *GRN* disease (Dopper et al., 2014, Premi et al., 2021, Premi et al., 2019). Interestingly, when clinical disease severity (CDR plus NACC score) was correlated with MBM eigenmodes, only the right-hemisphere mode 9 (frontal, temporal, and parietal pattern) reached significance. On the other hand, SBM analysis demonstrated a more widespread bilateral involvement. Thus, SBM and MBM demonstrated a complementary vision on the neuroanatomical underpinnings of disease progression, with SBM that captures local vertex-level aspects whereas MBM is more related to harmonic changes at network level. From this perspective, disease progression relies on the contralateral (right) hemispheric impairment, supported by local (SBM) and network (MBM) level breakdown, consistent with the pathological spreading observed in the advanced stages of the disease (Warren et al., 2013b).

Several limitations should be acknowledged. First, the small sample size may have limited our ability to detect significant effects in presymptomatic *GRN* carriers. Furthermore, even among symptomatic patients, the cohort size constrained our capacity to stratify subjects by clinical phenotype (e.g., bv FTD vs. PPA) and the entire sample by *GRN* mutation variants. Moreover, cognitive assessment was available only for symptomatic patients and examined limited cognitive domains: the lack of correlation between asymmetry indexes and cognitive features likely reflects the restricted cognitive domain assessed and/or a mismatch between the regions driving hemispheric asymmetry (notably posterior temporal and parietal cortices) and the predominantly frontal executive processes tapped by the TMT. From this perspective, future studies incorporating comprehensive assessments of language and other cognitive functions across both pre-symptomatic and symptomatic stages will allow a more precise evaluation of the sensitivity of SBM/MBM to cognitive decline. Finally, while the MBM approach is limited to cortical regions and does not capture subcortical structures such as the amygdala and hippocampus, which are also affected in FTS, it provides a tool to characterize multiscale cortical alterations, including subtle changes in presymptomatic individuals. Nevertheless, this study represents the first application of MBM to presymptomatic GRN mutation carriers.

In conclusion, applying a multiscale method such as MBM to conventional cortical thickness data revealed a more complex pattern of cortical involvement in symptomatic *GRN*-related disease, particularly within the left hemisphere. By leveraging information derived from specific resonant modes of the cortex—ranging from global to focal—we identified significant associations with cognitive performance, reflecting the network-based disruption that characterizes *GRN* disease and providing additional insights into the neuroanatomical substrates of cognitive impairment. Notably, MBM provided complementary information compared to SBM, also confirming the involvement of the contralateral hemisphere (and the corresponding reduction in asymmetry) as a distinctive feature of disease progression in *GRN*-related neurodegeneration. From this perspective, brain asymmetry remains a core hallmark of *GRN*-related pathology, supporting the use of asymmetry indices (derived from both SBM and MBM) as potential markers of disease progression. Further studies with larger samples and across different genetic forms of FTD are warranted to extend the application of this multiscale approach to cortical thickness analysis in the broader neurodegenerative field.

## CRediT authorship contribution statement

**Enrico Premi:** Writing – original draft, Methodology, Investigation, Data curation, Conceptualization. **Giada Bianchetti:** Writing – original draft, Visualization, Software, Methodology, Investigation, Formal analysis, Data curation. **Valeria Bracca:** Visualization, Investigation, Formal analysis, Data curation. **Giulia Campana:** Writing – original draft. **Elena Gatti:** Visualization, Investigation, Formal analysis. **Trang Cao:** Writing – review & editing, Software, Methodology. **Alex Fornito:** Writing – review & editing, Software, Methodology. **Valentina Cantoni:** Writing – review & editing. **Sonia Bellini:** Writing – review & editing. **Daniele Corbo:** Writing – review & editing, Methodology. **Mauro Magoni:** Writing – review & editing. **Roberto Gasparotti:** Writing – review & editing, Methodology. **Roberta Ghidoni:** Writing – review & editing, Validation, Supervision, Resources, Project administration, Funding acquisition, Conceptualization. **Michela Pievani:** Writing – original draft, Validation, Supervision, Software, Resources, Project administration, Investigation, Funding acquisition, Formal analysis, Conceptualization. **Barbara Borroni:** Writing – review & editing, Validation, Supervision, Resources, Project administration, Funding acquisition, Conceptualization.

## Declaration of competing interest

The authors declare that they have no known competing financial interests or personal relationships that could have appeared to influence the work reported in this paper.

## Data Availability

The datasets generated and/or analysed during the current study are available from the corresponding author on reasonable request. The dataset is publicly available at the following DOI: 10.5281/zenodo.17530608.

## Appendix A Supplementary data

Supplementary Data 1 Supplementary materials include surface-based morphometry (SBM) results, the symmetric bilateral mask for cortical thickness-based asymmetry index extraction, the correlation-based SBM asymmetry index, and effect size estimates (Cohen's d) for all pairwise comparisons.
