# Supplementary material for: Mapping the multiscale neuroanatomy of GRN-related frontotemporal dementia using mode-based morphometry
Source: Neuroimage Clin. 2026 Apr 15;50:103994. doi: 10.1016/j.nicl.2026.103994 (PMC13101774; doi:10.1016/j.nicl.2026.103994)
Supplement: Supplementary Data 1 — Supplementary materials include surface-based morphometry (SBM) results, the symmetric bilateral mask for cortical thickness-based asymmetry index extraction, the correlation-based SBM asymmetry index, and effect size estimates (Cohen's d) for all pairwise comparisons. [file mmc1.docx]

**Supplementary Materials**


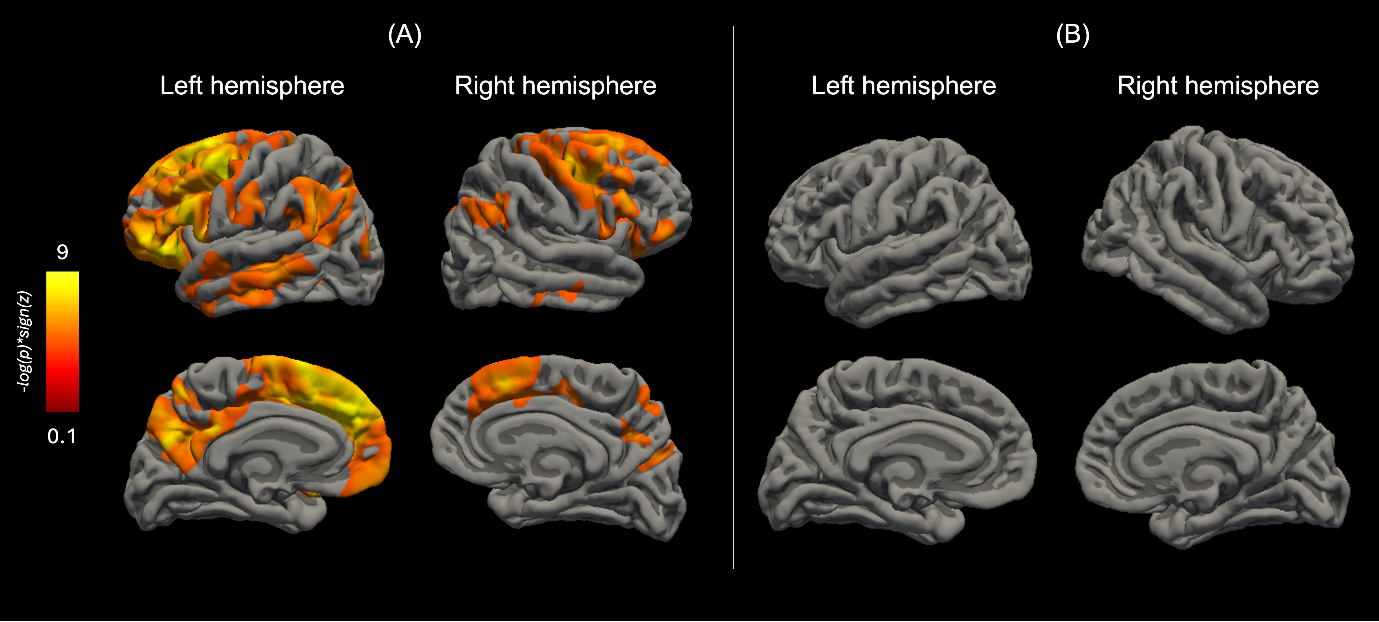


**Supplementary Figure 1.** Surface-based morphometry (SBM) results of CT contrasts for LH and RH. (A) *GRN* symptomatic carriers vs HC showed significant cortical thinning predominantly in the LH, with clusters involving frontal, parietal, and temporal lobes. (B) presymptomatic carriers vs HC did not show significant CT differences. Analyses used 10 FWHM-mm smoothing, a vertex-wise cluster-forming threshold of *p* < 0.001, and cluster-wise correction at *p* < 0.05. The color bar represents –log(*p*) values, with warmer colors (yellow) indicating stronger statistical significance.

**
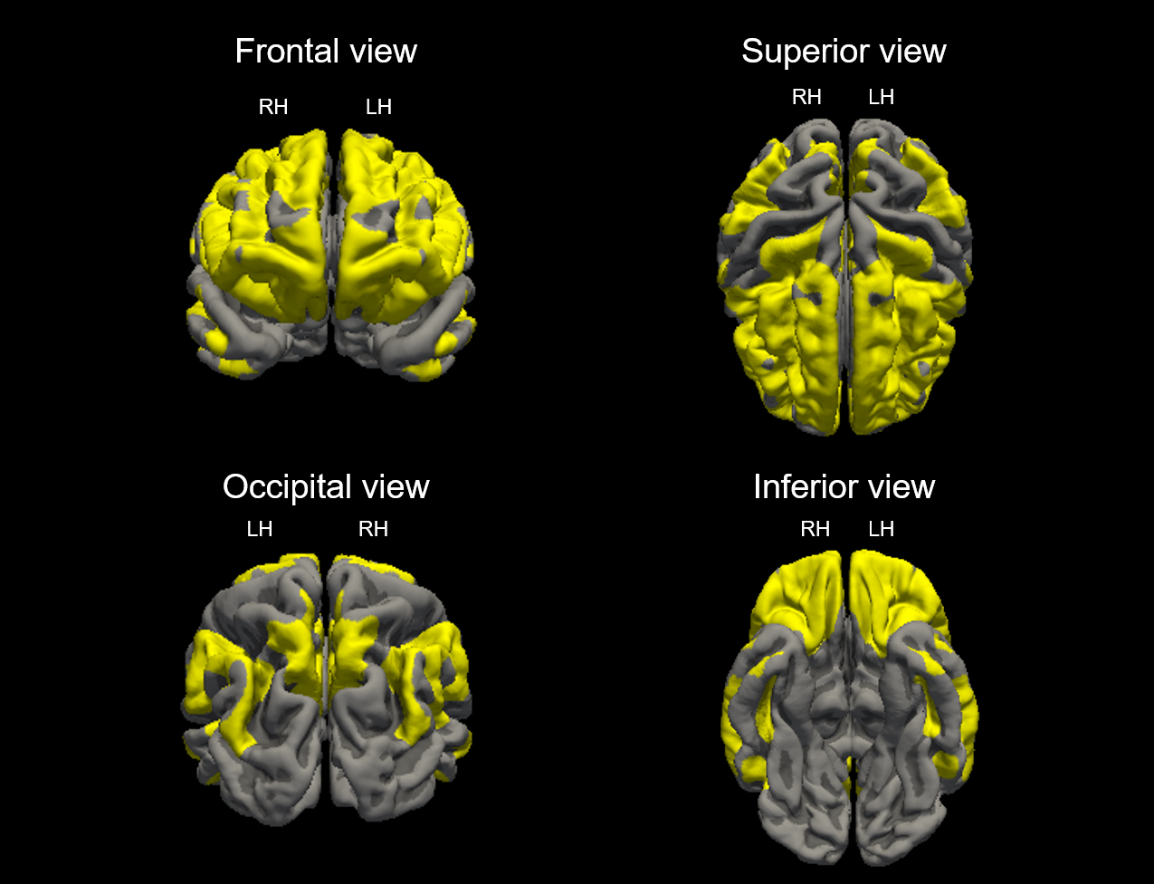
**

**Supplementary Figure 2. Symmetric bilateral mask for cortical thickness-based asymmetry index extraction.** The LH significance mask from the symptomatic *GRN* carriers’ vs HC contrast was thresholded (*p* < 0.05), binarized (yellow) and mirrored to the RH using FreeSurfer’s cross-hemispheric mapping. The symmetric bilateral region of interest was used to extract mean CT values from both hemispheres for conventional asymmetry index computation.

**Correlation-based SBM Asymmetry Index**

To assess whether the choice of metric influenced SBM-derived asymmetry indices, we also computed a correlation-based SBM-AI analogous to the MBM-AI. For each subject, CT values across the symmetric bilateral ROI (Supplementary Figure 2) were extracted for left and right hemispheres. The Pearson correlation between these vectors was computed and the absolute value taken to obtain an asymmetry index. Lower values indicate greater hemispheric dissimilarity, consistent with the convention SBM-AI interpretation.

Group-level comparisons using the Kruskal-Wallis test (*p* = 1.56×10⁻^6^) followed by Dunn’s post hoc test (with Benjamini-Hochberg correction), which are shown in Supplementary Figure 3, revealed that correlation-based SBM-AI values did not differ significantly between HC and presymptomatic individuals (*p*-adj = 0.892), whereas HC vs symptomatic (*p*-adj = 3.54×10⁻⁵) and presymptomatic vs symptomatic (*p*-adj = 3.68×10⁻⁴) differences were highly significant, indicating increased asymmetry in symptomatic *GRN* mutation carriers.


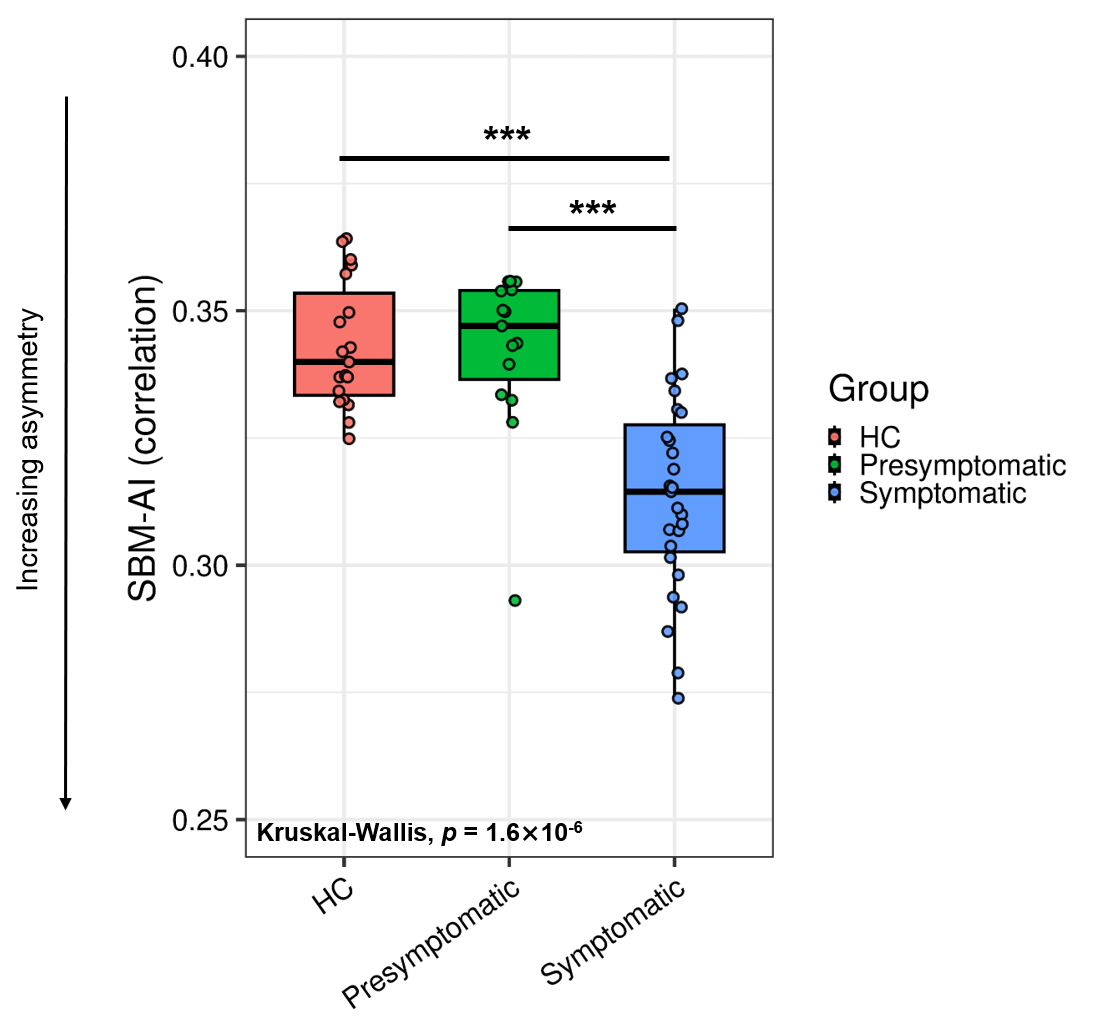


**Supplementary Figure 3. Correlation-based SBM Asymmetry Index across groups.** Boxplots show the distribution of correlation-based SBM-AI values for healthy controls (HC, in red), presymptomatic *GRN* mutation carriers (in green), and symptomatic *GRN* mutation carriers (in blue). Lower SBM-AI values indicate greater hemispheric asymmetry. Statistical differences between groups were assessed using Kruskal-Wallis test, with post-hoc comparisons indicated by asterisks (*** stands for *p* < 0.001).

**Supplementary Table 1. Effect size estimates (Cohen’s d) for MBM-AI vs Group.**

| **Group 1** | **Group 2** | **N_1_** | **N_2_** | **estimate** | **CI_lower** | **CI_upper** | **p-value** |
| --- | --- | --- | --- | --- | --- | --- | --- |
| Symptomatic | Presymptomatic | 27 | 15 | -1.5310 | -2.2642 | -0.7978 | < 0.0001 |
| Symptomatic | HC | 27 | 19 | -1.4777 | -2.1563 | -0.7990 | < 0.0001 |
| Presymptomatic | HC | 15 | 19 | 0.1392 | -0.5651 | 0.8436 | 0.9454 |

**Supplementary Table 2. Effect size estimates (Cohen’s d) for SBM-AI vs Group.**

| **Group 1** | **Group 2** | **N_1_** | **N_2_** | **estimate** | **CI_lower** | **CI_upper** | **p-value** |
| --- | --- | --- | --- | --- | --- | --- | --- |
| Symptomatic | Presymptomatic | 27 | 15 | -2.5106 | -3.3651 | -1.6562 | < 0.0001 |
| Symptomatic | HC | 27 | 19 | -2.6923 | -3.5195 | -1.8651 | < 0.0001 |
| Presymptomatic | HC | 15 | 19 | -0.1716 | -0.8764 | 0.5333 | 0.8642 |

**Supplementary Table 3. Effect size estimates (Cohen’s d) for MBM-AI vs CDR Global Score.**

| **Group 1** | **Group 2** | **N_1_** | **N_2_** | **estimate** | **CI_lower** | **CI_upper** | **p-value** |
| --- | --- | --- | --- | --- | --- | --- | --- |
| 2 | 0 | 11 | 10 | -2.9913 | -4.3215 | 1.661 | < 0.0001 |
| 2 | 0.5 | 11 | 6 | -1.1228 | -2.2798 | 0.0342 | 0.0782 |
| 2 | 3 | 11 | 3 | -1.9353 | -3.5628 | -0.3077 | 0.022 |
| 2 | 0_HC | 11 | 19 | -2.9283 | -4.0246 | -1.8319 | < 0.0001 |
| 2 | 1 | 11 | 7 | -0.459 | -1.4967 | 0.5787 | 0.2854 |
| 0 | 0.5 | 10 | 6 | 1.6255 | 0.358 | 2.893 | 0.0559 |
| 0 | 3 | 10 | 3 | 1.2855 | -0.266 | 2.8369 | 0.1119 |
| 0 | 0_HC | 10 | 19 | 0.1244 | -0.6779 | 0.9268 | 1 |
| 0 | 1 | 10 | 7 | 1.3277 | 0.1706 | 2.4848 | 0.0136 |
| 0.5 | 3 | 6 | 3 | -0.6951 | -2.4114 | 1.0213 | 0.7143 |
| 0.5 | 0_HC | 6 | 19 | -1.4947 | -2.5575 | -0.4318 | 0.0208 |
| 0.5 | 1 | 6 | 7 | 0.317 | -0.9152 | 1.5491 | 0.9452 |
| 3 | 0_HC | 3 | 19 | -0.7388 | -2.0554 | 0.5778 | 0.1597 |
| 3 | 1 | 3 | 7 | 0.6918 | -0.939 | 2.3226 | 0.3833 |
| 0_HC | 1 | 19 | 7 | 1.461 | 0.4572 | 2.4648 | 0.0103 |

**Supplementary Table 4. Effect size estimates (Cohen’s d) for SBM-AI vs CDR Global Score.**

| **Group 1** | **Group 2** | **N_1_** | **N_2_** | **estimate** | **CI_lower** | **CI_upper** | **p-value** |
| --- | --- | --- | --- | --- | --- | --- | --- |
| 2 | 0 | 11 | 10 | -1.9291 | -3.0356 | -0.8225 | 0.0037 |
| 2 | 0.5 | 11 | 6 | -0.3562 | -1.4457 | 0.7334 | 0.4043 |
| 2 | 3 | 11 | 3 | 0.3489 | -1.0775 | 1.7753 | 0.7692 |
| 2 | 0_HC | 11 | 19 | -2.5346 | -3.56 | -1.5091 | 0.0004 |
| 2 | 1 | 11 | 7 | 0.3611 | -0.6718 | 1.394 | 0.536 |
| 0 | 0.5 | 10 | 6 | 2.1414 | 0.7681 | 3.5147 | 0.011 |
| 0 | 3 | 10 | 3 | 6.2202 | 3.1693 | 9.2711 | 0.007 |
| 0 | 0_HC | 10 | 19 | -0.4294 | -1.2393 | 0.3805 | 0.6357 |
| 0 | 1 | 10 | 7 | 3.4592 | 1.8154 | 5.103 | 0.0002 |
| 0.5 | 3 | 6 | 3 | 0.9695 | -0.7877 | 2.7267 | 0.2619 |
| 0.5 | 0_HC | 6 | 19 | -3.1435 | -4.4792 | -1.8077 | 0.0044 |
| 0.5 | 1 | 6 | 7 | 0.8757 | -0.4059 | 2.1572 | 0.1375 |
| 3 | 0_HC | 3 | 19 | -12.3123 | -16.3953 | -8.2293 | 0.0013 |
| 3 | 1 | 3 | 7 | 0.0118 | -1.5795 | 1.6031 | 0.8333 |
| 0_HC | 1 | 19 | 7 | 4.8657 | 3.2007 | 6.5306 | < 0.0001 |
